# Flow Field Reconstruction and Prediction of Powder Fuel Transport Based on Scattering Images and Deep Learning

**DOI:** 10.3390/s25154613

**Published:** 2025-07-25

**Authors:** Hongyuan Du, Zhen Cao, Yingjie Song, Jiangbo Peng, Chaobo Yang, Xin Yu

**Affiliations:** 1 National Key Laboratory of Laser Spatial Information, Harbin Institute of Technology, Harbin 150001, China; duhongyuan2024@163.com (H.D.); songyingjie1117@163.com (Y.S.); pengjiangbo@hit.edu.cn (J.P.); yangchaobo@hit.edu.cn (C.Y.); yuxin0306@hit.edu.cn (X.Y.); 2 Postdoctoral Research Station of Power Engineering and Engineering Thermophysics, Harbin Institute of Technology, Harbin 150001, China

**Keywords:** scattering image, feature extraction, temporal prediction, stacked autoencoder, LSTM network

## Abstract

This paper presents the flow field reconstruction and prediction of powder fuel transport systems based on representative feature extraction from scattering images using deep learning techniques. A laboratory-built powder fuel supply system was used to conduct scattering spectroscopy experiments on boron-based fuel under various flow rate conditions. Based on the acquired scattering images, a prediction and reconstruction method was developed using a deep network framework composed of a Stacked Autoencoder (SAE), a Backpropagation Neural Network (BP), and a Long Short-Term Memory (LSTM) model. The proposed framework enables accurate classification and prediction of the dynamic evolution of flow structures based on learned representations from scattering images. Experimental results show that the feature vectors extracted by the SAE form clearly separable clusters in the latent space, leading to high classification accuracy under varying flow conditions. In the prediction task, the feature vectors predicted by the LSTM exhibit strong agreement with ground truth, with average mean square error, mean absolute error, and r-square values of 0.0027, 0.0398, and 0.9897, respectively. Furthermore, the reconstructed images offer a visual representation of the changing flow field, validating the model’s effectiveness in structure-level recovery. These results suggest that the proposed method provides reliable support for future real-time prediction of powder fuel mass flow rates based on optical sensing and imaging techniques.

## 1. Introduction

Powdered fuels, including high-energy metals and boron, are introduced into the combustion chamber in a gas–solid two-phase flow form under the action of fluidizing gas, endowing powder-fueled engines with advantages such as thrust flexibility and multi-pulse ignition [1,2,3]. As key components of the propulsion system, the stability and controllability of the powder fuel supply process are directly linked to the high-efficiency operation of powder-fueled engines. Achieving precise measurement and dynamic characterization of the powder fuel mass flow rate is crucial for ensuring combustion stability and overall system performance. However, real-time measurement of mass flow rate remains highly challenging due to the pronounced non-uniformity and transient nature of gas–solid two-phase flow structures [4]. Conventional measurement methods, such as the gravimetric method and piston displacement-based approaches, are commonly employed. For example, Baker et al. [5] studied the mass flow of magnesium powder using a piston displacement conversion method. Yet in practice, transient oscillations generated during piston movement significantly affected measurement stability and had to be mitigated through multi-point averaging. Although these methods can deliver reasonable accuracy under steady-state conditions, they suffer from long response times, delayed feedback, and limited capability for online monitoring—rendering them unsuitable for dynamic or complex working conditions. These limitations highlight the urgent need for fast-response, non-intrusive, and spatially resolved sensing technologies to enable real-time acquisition and modeling of powder mass flow behavior.

Accordingly, various fuel properties were utilized for detecting the fuel supply, such as absorption and electrostatic characteristics [6,7,8]. Significant studies were also carried out to obtain data related to fuel mass flow rate, employing techniques such as microwave attenuation [9], cross-correlation [10], Doppler-based methods [11], and thermal methods [12]. Although these techniques have demonstrated certain applicability in specific scenarios, their use remains limited in scope. Most existing research has focused on measurements within pneumatic conveying pipelines [13,14], while relatively few studies have addressed the unique requirements of powder fuel supply systems in engine applications. Compared to gaseous fuels, liquid fuels, or dilute gas–solid two-phase flows, the injection of powdered fuels typically forms a high-speed, dense gas–solid flow with strong signal noise interference, rendering many traditional methods unsuitable for direct application in such complex environments. In contrast, optical imaging-based sensing offers non-intrusive, high-resolution monitoring of complex flow fields, particularly under dense flow conditions [15]. Moreover, the optical approach allows for fast information transmission through structured light or spatial modes [16]. These methods visualize key flow features such as particle distribution, concentration gradients, and local structural evolution, providing valuable input for downstream analysis and modeling. When combined with image processing and data-driven algorithms, vision-based sensing has made significant strides in fuel monitoring. For instance, Ögren et al. [17] developed a real-time vision system integrating high-speed imaging and processing to estimate fuel feed rates in combustion devices. Laser sheet imaging has also been used to infer pulverized fuel mass flow rates from airflow particle distributions [18]. With the rapid development of computer vision and machine learning technologies, data-driven tools have been used in flexible and diverse ways across the field of fluid dynamics, particularly for extracting salient, application-specific flow features [19,20,21,22,23]. Dynamic prediction and reconstruction of flow structures facilitate indirect mass flow estimation, offering a solid foundation for precision control in powder fuel systems.

Over the past few decades, researchers have attempted to understand and characterize combustion conditions using traditional geometric features, with most efforts focused on extracting signal intensity, flow field location, and structural information [24,25]. However, these statistical approaches often fail to generalize across conditions, limiting their ability to model complex flow fields. Recently, machine learning has gained traction in combustion research for its ability to extract representative features from complex data. These models can identify hidden patterns and adapt to diverse flow conditions, offering improved accuracy and efficiency over rule-based methods. This data-driven shift has enhanced the flexibility and scalability of flow field analysis. Hasti et al. [26] developed a Support Vector Machine (SVM) model with radial basis functions to detect lean blowout in a gas turbine combustor. Chen et al. [27] applied Principal Component Analysis (PCA) to extract two key features from combustion images, enabling abnormal state detection while reducing data redundancy. Though traditional machine learning performs well with small datasets, it struggles with the nonlinear, high-dimensional nature of combustion image data. Deep learning, as a subset of machine learning, improves modeling capacity by learning high-level features through multi-layer nonlinear mappings. Roncancio et al. [28] proposed a Planar Laser-Induced Fluorescence (PLIF) image classification model using Convolutional Neural Networks (CNNs), achieving high accuracy and faster computation compared to conventional methods. However, CNNs require large labeled datasets, and data annotation remains labor-intensive. To overcome this, unsupervised models have been explored. Liu et al. [29] used a Deep Belief Network (DBN) to extract combustion features without labels. Akintayo et al. [30] employed a convolutional autoencoder (AE) for unsupervised combustion image representation, enabling stability assessment. In predictive tasks, Wang et al. [31] used a Deep Neural Network (DNN) to estimate particle velocity from swirl flow images. Han et al. [32] applied semi-supervised learning for combustion state prediction, while Tryambak et al. [33] developed a deep learning framework for lean blowout prediction. These advancements collectively highlight the potential of deep learning in enabling robust, label-efficient, and accurate modeling of complex flow dynamics. To systematically review the research progress and limitations of the aforementioned methods in mass flow rate characterization, Table 1 summarizes the representative approaches and categorizes them into two groups: direct measurement and feature-based estimation

Although extensive research has been conducted on applying machine learning techniques to extract features from combustion images, most of these efforts have focused on static classification tasks, such as combustion state identification or operational stability assessment [34,35,36]. These approaches generally lack the ability to capture the spatiotemporal information embedded in image sequences, making them less effective for dynamic feature prediction in real-time sensing scenarios. In this paper, we firstly established a high-speed optical imaging platform based on a gas–solid mixed combustion system. Using this setup, a large-scale dataset of boron particle scattering images was acquired under various carrier gas flow conditions. To preserve the essential structural characteristics of the flow field while significantly reducing computational complexity, appropriate preprocessing methods were then applied. Subsequently, a Stacked Autoencoder (SAE) was employed to perform unsupervised low-dimensional feature extraction from the images, effectively encoding the spatial structure of the flow field. The separability of the extracted features was validated using a Backpropagation (BP) neural network. These features were then organized into time-ordered sequences and fed into a Long Short-Term Memory (LSTM) network to enable dynamic prediction of future flow states. Finally, using the proposed SAE-BP-LSTM integrated framework, the flow field features could be sequentially modeled and the corresponding images could be reconstructed with high prediction accuracy and low reconstruction error. As an exploratory approach to flow field modeling and mass flow rate estimation, this framework is expected to integrate sensor-based perception and data-driven modeling simultaneously, enabling intelligent monitoring of powder fuel transport processes.

## 2. Experiment and Methodology Based on Deep Learning

### 2.1. Powder Fuel Supply System

**Figure 1:**
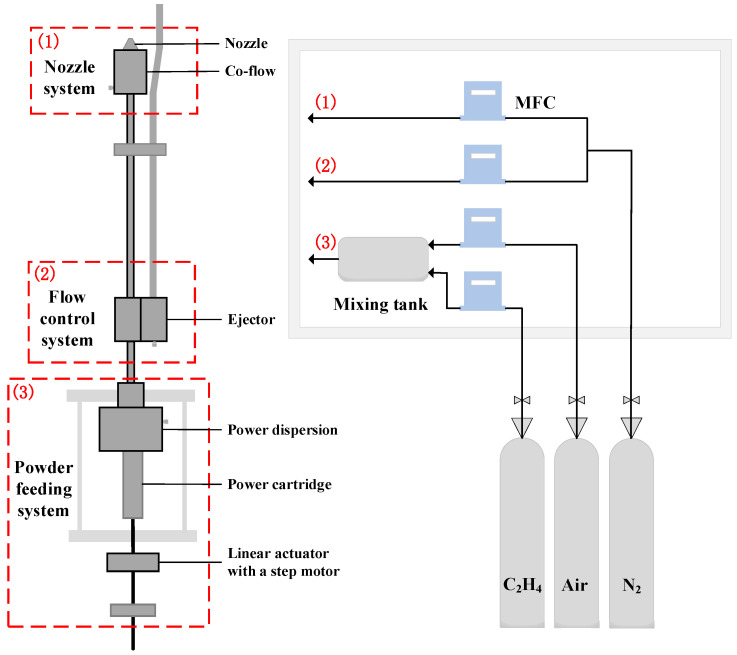
The structure of the powder fuel supply system.

The structural layout and external appearance of the gas–solid mixing burner employed in this study are shown in Figure 1. The system is composed of three functional subsystems: the nozzle and co-flow assembly, the flow regulation unit, and the powder dispersion module. At the heart of the design lies the powder dispersion mechanism, which plays a critical role in determining the efficiency of fuel delivery and the stability of the resulting two-phase flow. Specifically, the dispersion system leverages the shear force generated by an annular flow of premixed ethylene and air to entrain boron powder from the storage chamber and ensure thorough mixing downstream of the nozzle. This setup promotes both effective dispersion and uniform gas–solid mixing.

To enable precise control of the powder concentration, a stepper motor–driven piston is integrated into the fuel feed subsystem. This mechanism allows for accurate adjustment of the fuel delivery rate by controlling the piston’s speed. Ethylene, air, and nitrogen are each supplied from high-pressure gas cylinders, with their flow rates independently managed through high-precision mass flow controllers (MFCs). Real-time monitoring via flow meters ensures consistent gas composition, which is essential for maintaining stable combustion conditions.

**Figure 2:**
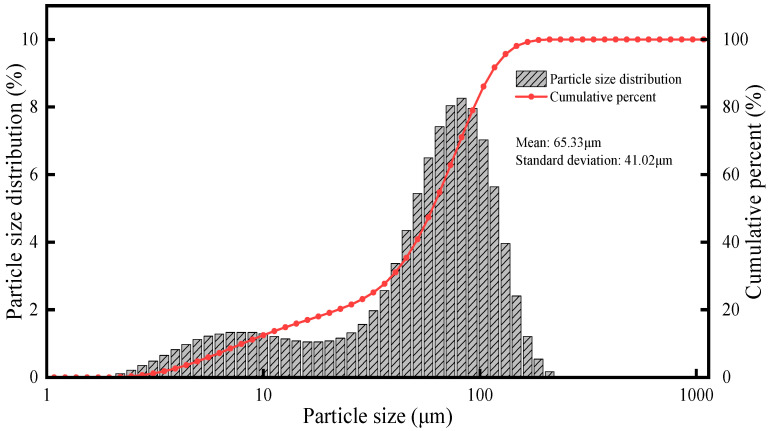
Particle size distribution of the boron powder, showing both volume histogram and cumulative curve.

In this paper, we selected boron powder as the fuel in this study due to its favorable performance characteristics and representativeness. The particle size distribution of the boron powder used in the experiments is illustrated in Figure 2. The distribution exhibits a wide range, with a weighted mean diameter of approximately 65.33 μm and a standard deviation of 41.02 μm, providing quantitative insight into the powder morphology.

To explore the correlation between flow field characteristics and fuel mass flow rate, a controllable transport strategy was developed. By modulating the combination of piston speed and carrier gas velocity, the mass flow rate of the powder fuel can be indirectly tuned. In this study, the piston speed was maintained at a constant value throughout all tests, while five distinct carrier gas flow rates were systematically configured to construct a range of transport conditions with varying mass flow rates. Table 2 lists the detailed experimental parameters corresponding to each condition. A gravimetric method was then employed to calibrate the actual powder flow rate under each condition, yielding reliable reference data. This calibration provides the foundation for subsequent image-based modeling and prediction of powder fuel mass flow rates.

### 2.2. Optical System

**Figure 3:**
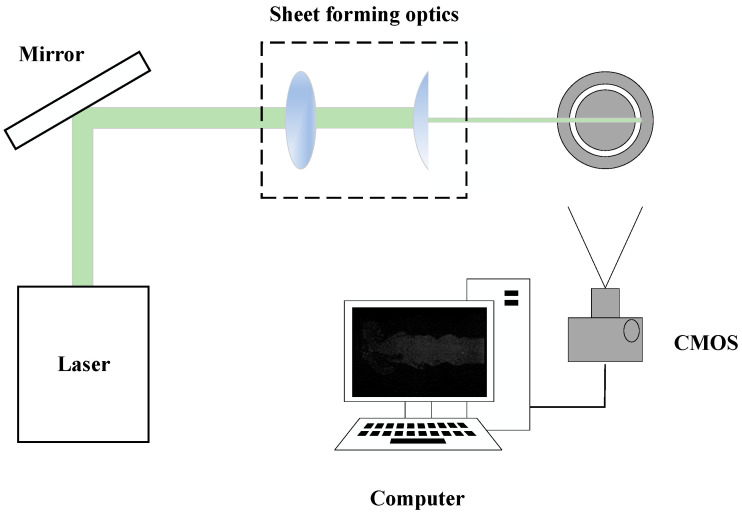
Schematic diagram of measurement system.

As illustrated in Figure 3, the optical diagnostic apparatus is composed of four main subsystems: a slice light formation system, an image acquisition module, a gas–solid two-phase mixing burner, and a synchronization-control unit. Together, these components enable high-fidelity visualization and recording of complex flow structures in powder-fueled combustion environments. The laser source employed in the system is a 10 kHz Nd: YAG laser (Edgewave, Germany), which emits a highly stable and coherent beam at a wavelength of 532 nm and a single-pulse energy of 10 mJ. This high-frequency pulsed output is particularly suitable for capturing transient flow phenomena. The slice light formation system consists of a set of optical elements including a concave lens, a collimating lens, and a focusing lens. These components transform the initially collimated laser beam into an elongated rectangular light sheet, with the shape of the front as a plane wave. The use of focusing lenses and apertures controls the beam’s geometric dimensions and directs it precisely into the measurement region. After passing through the optical train, the resulting light sheet reaches a height of approximately 9 cm and a thickness of about 0.5 mm, effectively illuminating the target flow field and enhancing the spatial resolution for imaging. The image acquisition system features a high-speed CMOS camera (pco.dimax HS, Kelheim, Germany), which captures sequences of scattering images at microsecond-scale intervals. The camera is oriented to record the motion and distribution of tracer particles within a defined measurement volume, typically 60 mm × 40 mm. All recorded images are digitized and stored in real time on a connected workstation for subsequent processing and analysis, such as feature extraction, flow reconstruction, or machine learning-based prediction.

To improve the fidelity of acquired images and reduce interference from ambient and stray light, a narrow-band optical filter is installed immediately in front of the camera lens. This selective filter allows only the laser wavelength to pass through, thereby significantly enhancing the signal-to-noise ratio (SNR). In addition, a synchronization-control system governs the precise timing of laser pulses and camera exposures. This hardware-level coordination ensures that both systems operate in perfect temporal alignment, maximizing the consistency and quality of the captured data across high-speed imaging sequences.

### 2.3. Dataset

**Figure 4:**
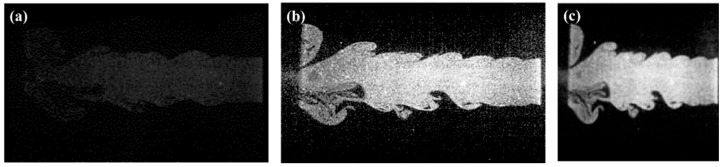
Image preprocessing steps for combustion radiation data: (**a**) original high-resolution image (1000 × 680); (**b**) contrast-enhanced image via histogram equalization; (**c**) resolution-compressed image (128 × 128) using linear combination method.

A specialized dataset was constructed using the aforementioned experimental apparatus. In this setup, the gas flow rate was adjusted to create five distinct flow conditions. For each condition, flow field structures were captured using a high-speed camera operating at 10 kHz, with each image recorded in a 1000 × 680 pixel format. A total of 1000 images were collected for each condition, yielding 5000 flow field scattering images in total. Before initiating model training, each image underwent contrast enhancement to better highlight the flow field configuration. As shown in Figure 4, the raw image (a) was first subjected to histogram-based contrast enhancement (b), improving the visibility of fine structural details [37]. To preserve the essential spatial structure while minimizing computational cost, the image resolution was reduced to 128 × 128 pixels (c) using bilinear interpolation [38] to preserve spatial structure while reducing computational cost. This preprocessing step, despite altering the original aspect ratio, significantly lowered the computational burden and improved the efficiency of subsequent analyses. It is worth noting that the input resolution was selected as an optimal trade-off between prediction accuracy and computational efficiency, based on empirical evaluation of model performance.

**Figure 5:**
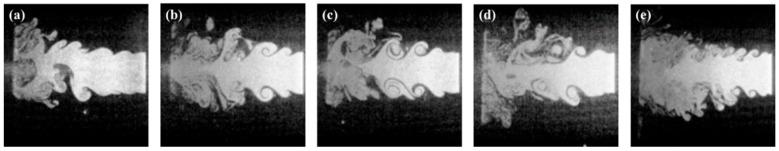
Representative scattering images of flow field structures captured under five different flow rate conditions: (**a**) 20 L/min, (**b**) 30 L/min, (**c**) 35 L/min, (**d**) 40 L/min, and (**e**) 50 L/min.

After preprocessing, typical flow structures observed under different flow rate conditions are shown in Figure 5, revealing distinct combustion morphology and particle distribution patterns across varying flow regimes. A differentiated data-splitting strategy was implemented for image datasets to accommodate the distinct requirements of static feature extraction and dynamic sequence modeling tasks associated with the characterization of flow-field structures, aiming to improve both training efficiency and model performance. In the static feature extraction phase, the original image data from each operating condition were randomly partitioned into training and testing subsets using an 8:2 ratio. The training subset was utilized for unsupervised feature learning, thereby deriving low-dimensional, discriminative representations. Conversely, the testing subset served as an objective basis to evaluate the clustering effectiveness and classification accuracy of the extracted features. For the dynamic sequence modeling task, the static features previously extracted were serialized into consecutive feature sequences aligned chronologically, with every five consecutive frames constituting one sequence sample. This approach resulted in the generation of 990 valid sequence samples per operating condition. These sequence samples were subsequently divided into training and testing sets using the same ratio, thereby providing abundant temporal context to the dynamic sequence model. The detailed dataset partitioning for both tasks is summarized in Table 3, which outlines the data forms, respective sample counts, and allocation percentages across training and test sets. This structured approach ensures that the model effectively captures the temporal evolution trends of the flow-field features, laying a solid foundation for subsequent feature prediction and image reconstruction tasks.

### 2.4. Deep Learning Methodology

**Figure 6:**
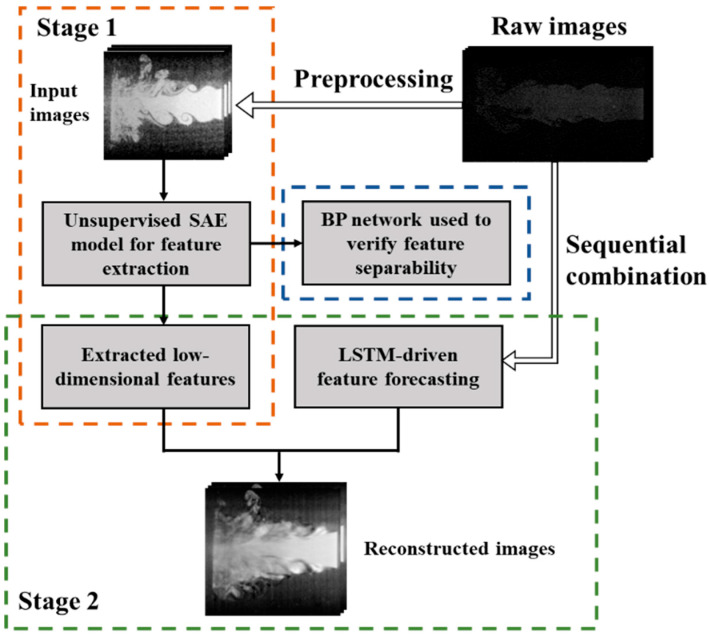
Schematic overview of the proposed deep learning strategy for flow-field image modeling.

To provide a clear and structured overview of the proposed modeling strategy, the overall deep learning framework is conceptually illustrated in Figure 6. This schematic highlights the integration of static feature extraction and dynamic sequence modeling, serving as a roadmap for the subsequent technical development. The methodology combines SAE for unsupervised feature extraction, BP for static classification, and LSTM for temporal prediction. Within this framework, BP is employed to evaluate the quality and discriminative capability of the features extracted by SAE, while LSTM models the temporal evolution of the encoded representations. This modular design enables targeted evaluation of both spatial and temporal components and facilitates a more interpretable analysis of intermediate representations.

The modeling process begins with SAE, which serves as the foundational feature extraction module [39]. As a symmetrical neural network trained in an unsupervised manner, SAE is designed to learn compact latent representations by minimizing the reconstruction error between the input and output images. It consists of multiple encoding and decoding layers, where the encoder compresses high-dimensional grayscale input images into low-dimensional feature vectors, and the decoder attempts to reconstruct the original input [40]. This structure allows the model to preserve essential spatial features of the flow field while reducing data dimensionality.

To evaluate whether the extracted features are sufficiently discriminative, a BP neural network is introduced for static classification. Acting as a supervised validation stage, the BP network maps the latent features to their corresponding flow conditions. It consists of multiple hidden layers activated by nonlinear functions and is optimized using labeled data to minimize classification error [41]. The classification accuracy in this stage provides a measure of how well the SAE captures regime-specific characteristics within its learned feature space.

For sequence modeling, an LSTM network is incorporated to capture the temporal dynamics of the flow field. Unlike feedforward networks, LSTM maintains an internal memory state that enables it to learn long-range dependencies across input sequences [42]. In this framework, the LSTM receives a sequence of consecutive latent feature vectors as input and is designed with two output branches: one predicts the feature vector of the next frame, and the other reconstructs the future image through the decoder of the SAE. By jointly optimizing both prediction and reconstruction objectives, the network is trained to model the temporal evolution of both internal representations and external visual structures.

**Figure 7:**
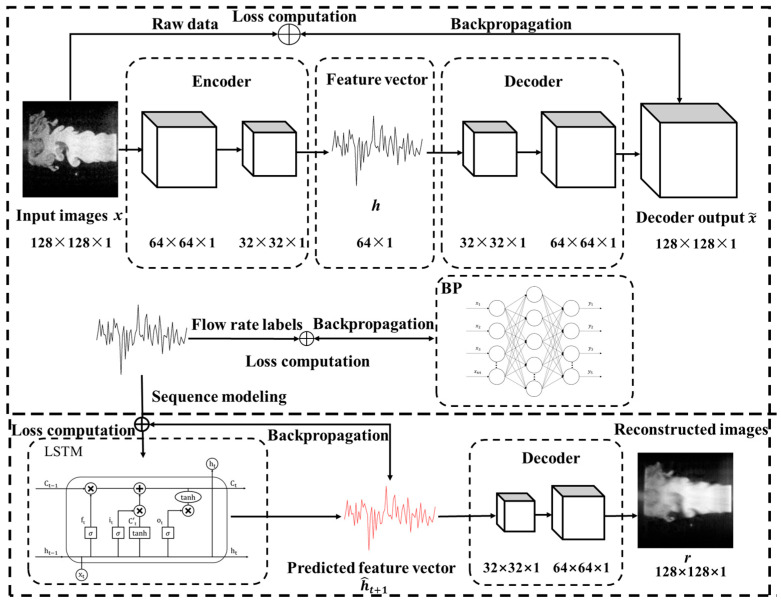
Implementation details and training workflow of the proposed deep learning architecture.

The technical realization of the proposed framework is elaborated in Figure 7, which illustrates the internal structure of each module and outlines the corresponding training workflow adopted to ensure effective implementation and integration of the overall system. The SAE first encodes the input image $x\in R^{128\times 128}$ into a 64-dimensional latent vector *h* through multiple encoding layers, with the entire network optimized via backpropagation to enhance its ability to extract structural features from the flow field. These learned features are then utilized in two parallel branches. In the first branch, *h* is directly fed into a BP neural network, which is trained in a supervised fashion using labeled mass flow rate data. This enables the model to learn discriminative mappings from latent feature vectors to their corresponding flow regimes. In the second branch, the extracted feature vectors are organized into a temporal sequence $\left\{h_{t}\right\}$, which is input into an LSTM network to predict the feature vector at the next time step ${\hat{h}}_{t+1}$. The predicted vector is then passed through a pretrained SAE decoder to reconstruct the corresponding flow field image *r*. This pathway enables the model to capture the temporal evolution of flow structures and generate accurate visual reconstructions of future flow field states.

All model training and evaluation procedures were conducted on a GPU-accelerated computing platform. The framework was implemented in Python 3.12 using the PyTorch 2.5.1 deep learning library. Image preprocessing was performed with OpenCV 4.11 and NumPy 1.26, and result visualizations were generated using Matplotlib 3.10 and Seaborn 0.13.

### 2.5. Performance Evaluation Indicators for the Models

A set of quantitative evaluation metrics was employed to systematically assess the model’s performance across both static classification and dynamic prediction tasks. These metrics facilitate rigorous comparisons at various stages of the modeling framework, ensuring robust validation of the proposed methods.

In terms of the static classification performance based on extracted features, four widely recognized indicators were selected to comprehensively assess model effectiveness. Specifically, Accuracy (*acc*) reflects the overall proportion of correct predictions; Precision (*p*) measures the proportion of true positives among all predicted positives; Recall (*r*) captures the model’s ability to identify all relevant samples of a given class; and the F_1_-score (*F*_1_), defined as the harmonic mean of precision and recall, provides a balanced assessment of model performance, especially under class-imbalanced scenarios. These metrics are formally defined as follows:

(1) $$\begin{array}{c} acc=\frac{TP+TN}{TP+TN+FP+FN} \end{array}$$

(2) $$\begin{array}{c} p=\frac{TP}{TP+FP} \end{array}$$

(3) $$\begin{array}{c} r=\frac{TP}{TP+FN} \end{array}$$

(4) $$\begin{array}{c} F_{1}=\frac{2\cdot p\cdot r}{p+r} \end{array}$$

where TP (True Positives), FP (False Positives), FN (False Negatives), and TN (True Negatives) represent the standard components of the confusion matrix [43].

Regarding the dynamic predictive performance associated with temporal modeling by the LSTM network, three conventional regression metrics were utilized: Mean Squared Error (MSE), Mean Absolute Error (MAE), and the Coefficient of Determination (R^2^). Among these, MSE and MAE quantify the average differences between the predicted and actual values. MSE is particularly sensitive to larger prediction errors, which aids in identifying potential outliers, while MAE offers an intuitive measure of overall prediction accuracy. In contrast, R^2^ evaluates how effectively the predicted features explain the variability observed in the true data, with values closer to 1 indicating superior model fitting. These metrics are mathematically expressed as follows:

(5) $$\begin{array}{c} MSE=\frac{1}{n}\sum_{i=1}^{n}(y_{i}-{\hat{y}}_{i}{)}^{2} \end{array}$$

(6) $$\begin{array}{c} MAE=\frac{1}{n}\sum_{i=1}^{n}\left|y_{i}-{\hat{y}}_{i}\right| \end{array}$$

(7) $$\begin{array}{c} R^{2}=1-\frac{\sum_{i=1}^{n}(y_{i}-{\hat{y}}_{i}{)}^{2}}{\sum_{i=1}^{n}(y_{i}-{\bar{y}}_{i}{)}^{2}} \end{array}$$

where $y_{i}$ denotes the actual feature values, ${\hat{y}}_{i}$ represents the predicted values, ${\bar{y}}_{i}$ is the mean of the true values, and n is the number of feature dimensions.

Collectively, these metrics offer a comprehensive, multi-dimensional evaluation framework for both classification accuracy and prediction precision, facilitating a robust assessment of the proposed modeling approach.

## 3. Results

### 3.1. Feature Extraction

**Figure 8:**
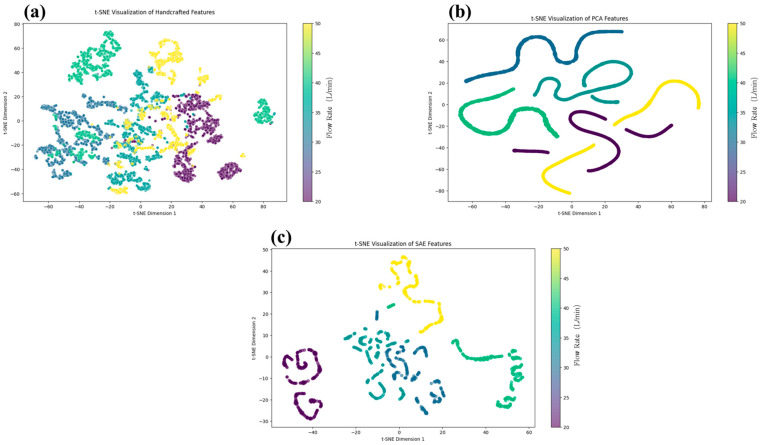
t-SNE visualization of extracted features using three methods: (**a**) Handcrafted geometric features; (**b**) PCA-based dimensionality-reduced features; (**c**) SAE-derived deep features.

In this study, to evaluate the representational capability of different feature extraction methods for flame flow field structures, we extracted features from the collected experimental images using three approaches: traditional handcrafted features (including area, perimeter, curvature, and centroid coordinates), PCA-based dimensionality-reduced features, and deep features derived from the SAE. The extracted features were subsequently visualized using t-distributed Stochastic Neighbor Embedding (t-SNE), a widely adopted technique for visualizing high-dimensional data by projecting them into a two-dimensional space, thereby revealing the intrinsic structure and clustering tendencies among samples. As shown in Figure 8, the t-SNE projections of handcrafted features (Figure 8a), PCA features (Figure 8b), and SAE-derived features (Figure 8c) offer a comparative perspective on the separability and compactness of each method’s output. In each sub-image, the data points are color-coded according to their corresponding flow rate condition (20, 30, 35, 40, and 50 L/min), as indicated by the adjacent color bar.

The t-SNE results demonstrate that while all three types of features exhibit some degree of class separability in the embedded space, the features extracted by the SAE show significantly better clustering performance compared to handcrafted or PCA features. Specifically, handcrafted features, though capable of capturing basic geometric attributes of the flame contours, are limited by their low dimensionality (only seven features) and strong dependence on domain knowledge. As a result, the distributions of different classes exhibit substantial overlap and ambiguous cluster boundaries. PCA, as a classical unsupervised linear dimensionality reduction method, improves feature separability to some extent; however, it retains only the principal linear components and fails to capture the underlying nonlinear patterns in the images, leading to indistinct cluster formation.

In contrast, SAE employs layer-wise nonlinear encoding to automatically extract multi-level abstract representations from high-dimensional image data. These representations are more effective at capturing deep features such as texture, structural variation, and intensity distribution. As shown in the t-SNE projection, SAE-extracted features yield well-separated and tightly clustered groups for most working conditions, indicating high intra-class compactness and inter-class separability. One notable exception occurs between the conditions at 30 L/min and 35 L/min, where partial overlap is observed in the projected feature space. Further inspection of the raw images suggests that these two conditions exhibit highly similar particle aggregation patterns, which may challenge the encoding model’s ability to fully discriminate between them.

Despite this localized ambiguity, the overall clustering structure of SAE features remains clear and robust, highlighting their superior representational and discriminative power. The deep feature modeling capability of SAE provides a solid foundation for downstream tasks such as mass flow rate prediction and temporal evolution modeling, confirming its effectiveness as an unsupervised feature extraction approach for combustion image analysis.

**Figure 9:**
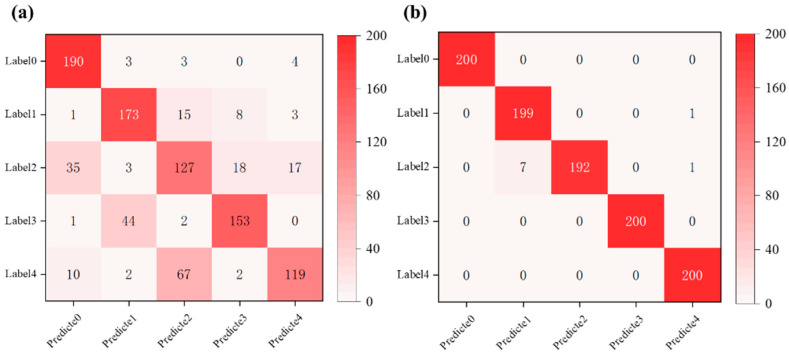
Confusion matrices of BP classifier using (**a**) handcrafted features and (**b**) SAE-derived features for flow condition classification.

To further validate the preliminary observations in a practical classification context, we conducted a comparative study using handcrafted features and SAE-derived features as inputs to a BP neural network for the classification of flow field images under different operating conditions. The resulting confusion matrices were used to assess the classification performance. The classification performance was visualized through confusion matrices, as shown in Figure 9, where Figure 9a corresponds to the manually engineered features and Figure 9b presents the results using SAE-extracted deep features. Although PCA-based features also demonstrated a certain level of accuracy, PCA, being a linear dimensionality reduction technique, lacks interpretability and the ability to capture deeper semantic structures. Therefore, this study focuses on the performance comparison between manually engineered features and those automatically learned by the SAE.

As illustrated by the confusion matrices, the classifier using SAE features exhibits significantly stronger discriminative capability across flow regimes, with the majority of predictions aligning along the diagonal. This indicates a high level of classification accuracy and robustness in identifying various flow conditions.

The SAE-based classification model achieved an overall test accuracy of 99.1%, indicating its strong capability in extracting semantically meaningful and discriminative features from high-dimensional scattering spectral images. These learned representations effectively capture the spatial intensity distribution differences under varying flow conditions, enabling the BP classifier to construct precise decision boundaries and perform accurate state recognition. For comparison, the classification accuracy obtained using handcrafted features was only 76.2%, indicating that the automatically learned features provide a significantly more informative and robust representation. Overall, the SAE–BP classification framework demonstrates promising applicability in combustion diagnosis and operational condition identification tasks.

### 3.2. Time Series Prediction

**Figure 10:**
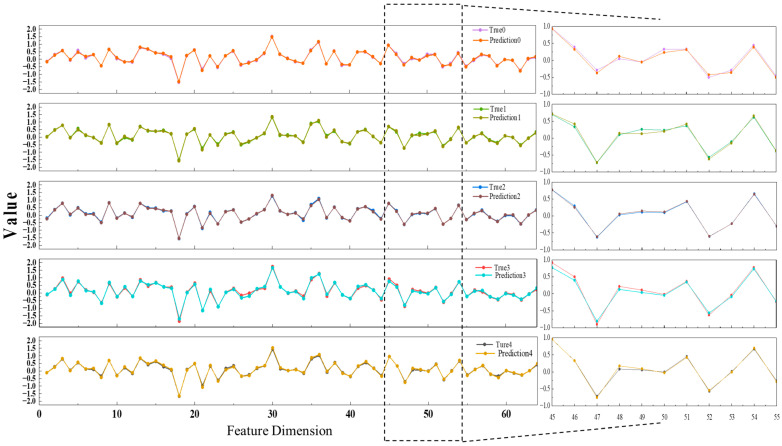
Comparison of predicted and ground-truth feature vectors across five flow regimes.

Under five distinct operating conditions, the constructed LSTM model was applied to forecast the evolution of image feature sequences and compare its predictions against the ground-truth feature vectors extracted by the SAE network. As shown in Figure 10, representative samples from each category were selected, and their 64-dimensional feature vectors were visualized as line plots to intuitively illustrate the correspondence between predicted and actual values across dimensions. A strong consistency was observed between the predicted features and the true trajectories, with numerical trends closely aligned. This reflects the model’s ability to effectively capture temporal dependencies and learn the evolving patterns of flow field structures. The consistency of these results across all five flow regimes further demonstrates the robustness and generalization capability of the proposed approach in feature-level time-series prediction.

Table 4 summarizes the predictive performance of the proposed model under five distinct flow conditions. The results indicate consistently low prediction errors and high fitting accuracy across all scenarios, with an average MSE of 0.0027, an MAE of 0.0398, and an average R^2^ of 0.9897. These metrics collectively demonstrate the model’s strong capability in accurately forecasting flow field features and its stable generalization performance across varying operating regimes.

**Figure 11:**
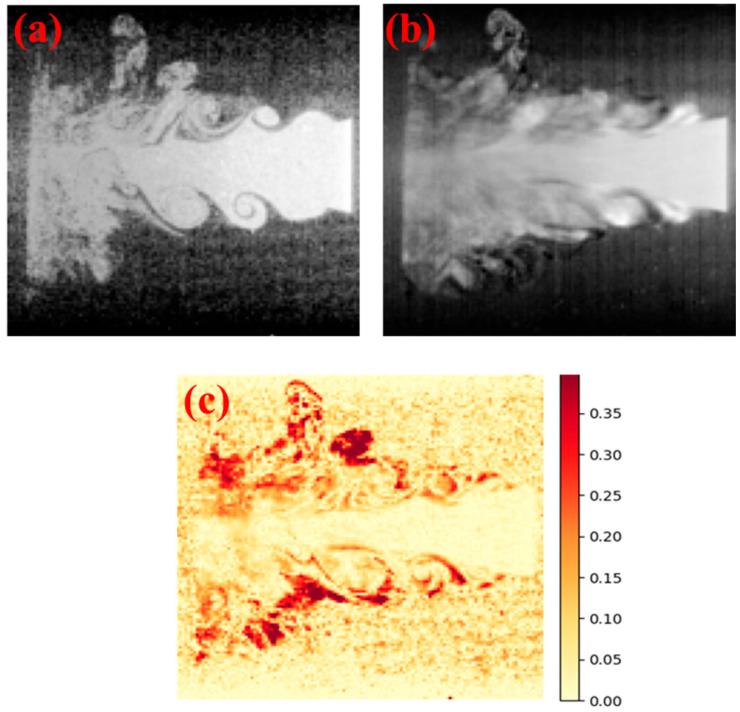
Visualization of image reconstruction results: (**a**) original image, (**b**) reconstructed image, and (**c**) pixel-wise error heatmap.

In addition, to evaluate the model’s effectiveness in reconstructing spatial image information, the 128-dimensional hidden state vectors produced by the LSTM were passed into the trained decoder of the SAE to generate the corresponding flow field images. As shown in Figure 11, the original image (Figure 11a), the reconstructed image (Figure 11b), and the pixel-wise error heatmap (Figure 11c) were visualized for representative samples. The reconstructed images exhibit a high degree of structural similarity to the ground truth, with only minor discrepancies observed in edge regions and areas of abrupt transition. The error heatmap further confirms that reconstruction errors remain minimal across most pixels, indicating the model’s strong ability to preserve and recover spatial structural information. Overall, the combined feature prediction and image reconstruction framework not only captures the temporal evolution of flow field features effectively but also offers a reliable technical basis for dynamic mass flow rate monitoring and visual interpretability.

## 4. Discussion

In this study, a deep learning-based joint modeling framework was developed to characterize and predict the evolution of flow fields during powder fuel transportation using high-frequency scattered images. One key advantage of this approach lies in the use of SAE, which can automatically learn latent spatial distribution structures from complex scattered images and extract low-dimensional feature vectors with high discriminative power. Given the significant nonlinearity of flow structures under different mass flow rates, it is difficult to accurately describe them using a limited set of geometric descriptors such as shape contours, spatial centroids, or projected areas. As a result, this method proves particularly effective in scenarios involving high noise, complex flow morphologies, and limited prior knowledge. In addition, the BP network classifies the feature vectors extracted by the SAE and effectively distinguishes between different flow rate conditions. This reflects a latent correlation between the extracted features and the underlying flow states. Furthermore, the LSTM network exhibits excellent temporal modeling capabilities. It retains long-term dependencies in image sequences, enabling the predicted feature vectors over time to match the ground truth across various evaluation metrics. This study establishes a fully data-driven alternative to traditional flow field analysis without relying on physical modeling or handcrafted features, addressing both state recognition and flow field prediction in the context of powder fuel transportation diagnostics. This approach is particularly suited to systems where flow structures are difficult to model and parameters are hard to observe, providing a transferable foundation for researchers to perform image-based analysis and feature modeling in other physical processes. It also facilitates further exploration of the intrinsic relationships between image-derived features and key physical parameters such as particle size, velocity, and mass flow rate.

It is worth noting that in the reconstructed images, regions that exhibit stronger pixel-wise heterogeneity, such as the boundaries between areas of high and low particle concentration or zones with steep gradients, tend to correspond to slightly elevated reconstruction errors. This may be attributed to the increased complexity and variability within these regions, which present greater challenges for accurate temporal feature prediction and decoding. This observation underscores an inherent limitation of sequential learning models in coping with abrupt spatiotemporal transitions. In such cases, even slight deviations in predicted features can be magnified during the decoding process, resulting in localized errors. Edge regions often encapsulate physically meaningful phenomena, including flow shear, particle clustering, or the onset of turbulence. These phenomena typically introduce high-frequency components into the image, which are more difficult to reconstruct from compressed representations. These findings indicate that future improvements could involve the integration of attention mechanisms or spatial refinement modules to suppress errors in critical regions. Alternatively, adopting a multi-scale training strategy that emphasizes fine-grained structural learning may enhance the model’s sensitivity to local spatial variations.

Importantly, the framework was designed using a modular architecture, separating feature extraction and temporal modeling instead of adopting a fully end-to-end approach. This design enhances training flexibility, enables independent performance assessment of submodules, and reduces overfitting risk caused by redundant information. Moreover, the modular structure provides a practical foundation for incorporating physical priors, such as particle size distribution or mass flow rate data, in subsequent developments.

## 5. Conclusions

The flow field structure is recognized as reflecting the distribution of fuel particles and the dynamic evolution of gas–solid flows, thereby providing a basis for analyzing the relationship between mass flow rate and flow behavior. Given its impact on engine thrust and safety, a practical framework for real-time flow field characterization is essential, and such a framework has been presented in this research paper.

In this work, a trained SAE operating in an unsupervised paradigm is employed to automatically capture discriminative features embedded in scattering images of powder-fuel flow structures. Compared with principal-component and handcrafted features, the SAE representations exhibit markedly tighter clusters and clearer inter-class boundaries, as confirmed by t-SNE visualization. To model the temporal evolution of these features for prediction and reconstruction, an LSTM network is introduced. Experiments conducted at five representative operating conditions yield an average MSE of 0.0027, MAE of 0.0398, and R^2^ of 0.9897; the reconstructed images faithfully preserve the structural details of the original flow fields, confirming the framework’s effectiveness in both feature prediction and visual recovery. Although the present validation is conducted under a limited set of operating conditions, the data-driven nature of the proposed architecture allows for straightforward extension to broader and potentially continuous flow scenarios. The proposed framework thus offers a sound theoretical and methodological basis for intelligent monitoring of powder-fuel transport and can be transferred to other complex thermophysical processes. Future work will integrate scattering images with gravimetrically calibrated mass-flow data to enable real-time online estimation and to elucidate intrinsic links between particle properties and delivery rates.

## Figures and Tables

**Table 1:** Summary of representative methods for mass flow rate characterization.

Category	Method	Advantages	Limitations
Direct measurement	Gravimetric method and piston displacement	Simple and accurate under steady conditions	Slow response, poor dynamic adaptability
	Microwave attenuation and Doppler method	Non-intrusive, capable of online monitoring	Limited accuracy in dense or noisy flow fields
Feature-based inference	Geometric and statistical features	Intuitive physical meaning, simple to implement	Poor generalization, limited to predefined scenarios
	Traditional machine learning approaches	Effective on small datasets, interpretable	Weak in capturing nonlinear, high-dimensional flow characteristics
	Deep learning approaches	Strong feature extraction, adaptable to complex flow conditions	Requires large datasets, high computational cost

**Table 2:** Operating conditions.

Case	Fuel	Air (L/min)
1	Boron powder	20
2		30
3		35
4		40
5		50

**Table 3:** Data Allocation for Model Training and Evaluation.

Purpose	Data Form	Training Set (80%)	Test Set (20%)	Total
Feature Extraction	Single-frame Image	4000	1000	5000
Time Series Prediction	Feature Sequence	3975	975	4950

**Table 4:** Quantitative evaluation of feature prediction performance under five flow conditions.

Index	Case 1	Case 2	Case 3	Case 4	Case 5
MSE	0.0037	0.0016	0.0015	0.0042	0.0026
MAE	0.0483	0.0310	0.0305	0.0490	0.0401
R^2^	0.9851	0.9933	0.9934	0.9873	0.9893

## Data Availability

Data are contained within the article.
